# Meningioangiomatosis Without Neurofibromatosis Type 2

**DOI:** 10.4021/wjon470w

**Published:** 2012-07-05

**Authors:** Sara Marzi, Danilo De Paulis, Alessandro Ricci, Graziano Taddei, Soheila Raysi Dehcordi, Gino Coletti, Giuliano Maselli, Renato J. Galzio

**Affiliations:** a Department of Neurosurgery, San Salvatore Hospital, L’Aquila, Italy; b Department of Pathology, San Salvatore Hospital, L’Aquila, Italy; c Department of Health Sciences, San Salvatore Hospital, L’Aquila, Italy

**Keywords:** Meningioangiomatosis, Cerebral hamartoma, Neurofibromatosis type 2

## Abstract

Meningioangiomatosis (MA) is a rare, benign hamartomatous lesion found in cerebral cortex and leptomeninges. It occurs mostly in 5 - 15 year old children in form isolated or diffuse; the diffuse form may be associated with neurofibromatosis type 2 (NF2). The sporadic type in the adults is less common.The patient was a 37 year-old man with a long history of frontal headache. In suspected sinusitis, the patient underwent cerebral MRI that showed hypointense lesion in the right frontal lobe with heterogeneous contrast enhancement after gadolinium administration. There were no stigmata or family history of neurofibromatosis. A right pterional approach with a supraorbital craniotomy was performed. The lesion was removed with complete remission of the headache in the postoperative time. MA enters into differential diagnosis with several other diseases and a correct diagnosis is mandatory. The total surgical removal is the treatment of choice, and the prognosis after surgery is usually excellent for the absence of recurrence in sporadic cases.

## Introduction

Meningioangiomatosis (MA) is a rare, benign hamartomatous lesion found in cerebral cortex and leptomeninges [1]. It is characterized by intracortical proliferation of meningothelial cells, microvasculature and fibroblasts that may mimic a neoplasm clinically and radiologically [2] MA may occur mostly in 5 - 15 year old children and may be isolated or diffuse; the diffuse form may be associated with neurofibromatosis type 2 (NF2) [3]. Sporadic MA usually presents seizures and persistent headaches, while NF2-associated forms are often asymptomatic and diagnosed only at autopsy [4]. The authors describe a case of sporadic form of MA unassociated with NF2 reviewing the pertinent literature.

## Case Report

**Figure 1:**
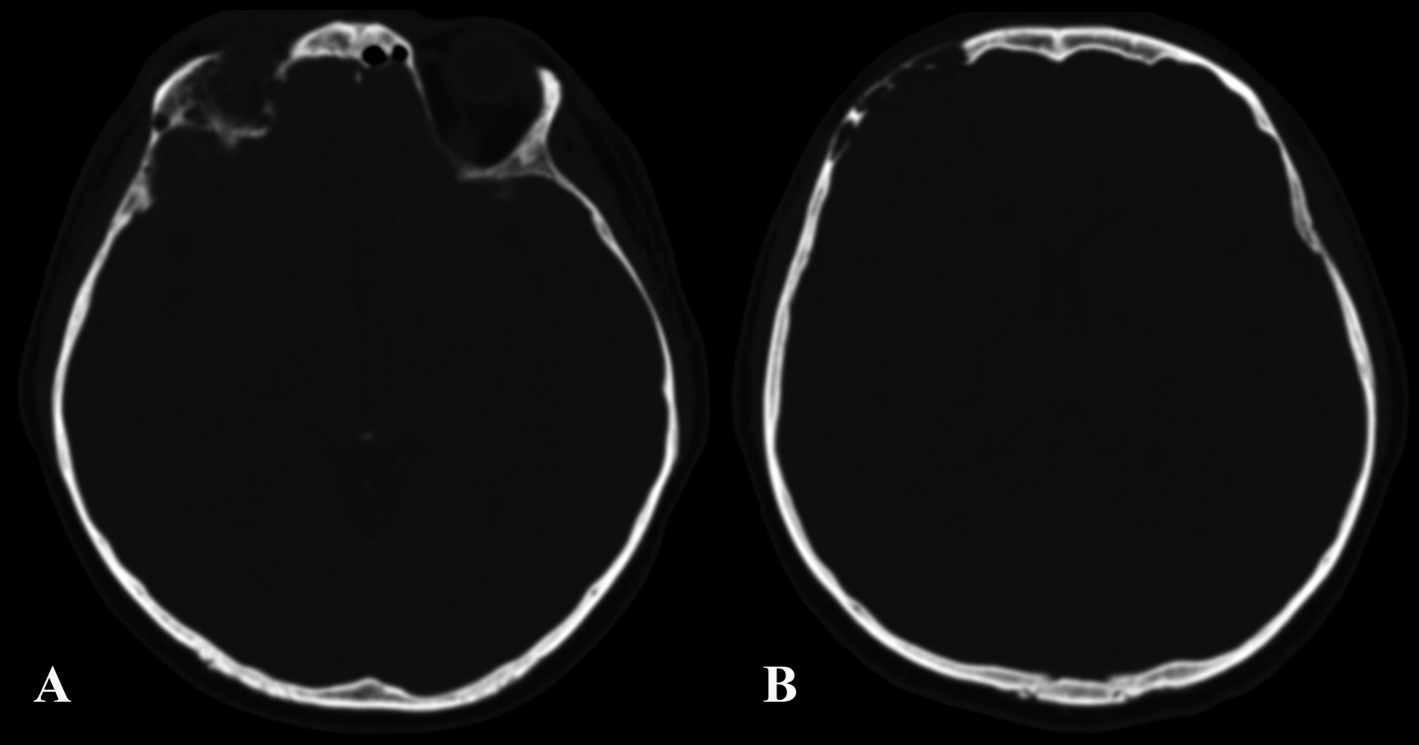
Pre-operative CT showing the involvement of the right orbital roof (A) and the right frontal bone (B) by the lesion.

**Figure 2:**
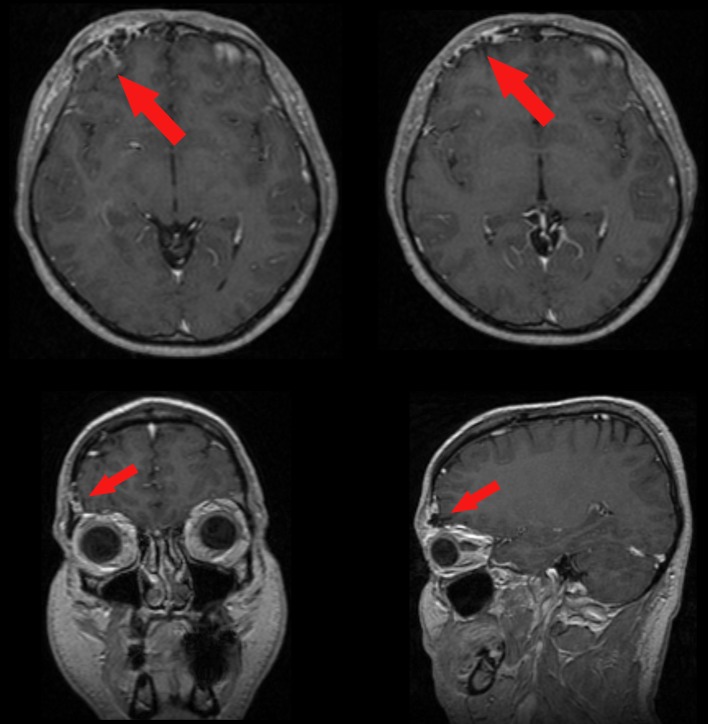
Pre-operative enhanced MR showing the meningioangiomatosis (red arrows) in the 3-plains projection.

A 37-year-old man was admitted to our hospital complaining of frequent episodes of headache for the past several months. He was followed by the otolaryngologist for sinusitis, but for the persistence of the headache, computed tomography (CT) and magnetic resonance (MR) of the brain were performed. CT scans showed a right frontal calcified lesion e (Fig. 1) and marked enhancement post contrast and MR revealed hypointense lesion in the right frontal lobe with heterogeneous contrast enhancement after Gadolinium administration (Fig. 2). On admission to our department, general, physical and neurological examination found no abnormalities. X-ray scan of the skull revealed an extended geographical area of osteolysis in charge of the frontal squama in the right supraorbital region with involvement of the roof of the ipsilateral orbit and hypoplasia of frontal sinus. A brain CT and MR were repeated and showed no changes beyond those already performed. There were no stigmata or family history of neurofibromatosis.

A right pterional approach was performed. The bone presented a gap full of blood sized 3 × 2.5 cm. The craniotomy was performed round this gap with a margin of 1 cm. The roof of the orbit ipsilateral was involved by the fibrous part of the lesion, which was not well demarcated from the surrounding soft brain parenchyma. We performed a progressive vascular deafferentation and disconnection of the lesion from the parenchyma to its anteromedial segment that appeared in proximity to the base of the superior sagittal sinus through the vein of Sperino. Dura appeared thickened, well vascularized and its dissection from the parenchyma was difficult. At the end, the alterated dural surface was replaced by a patch of galea and the bone reconstruction was obtained using his bony flap.

**Figure 3:**
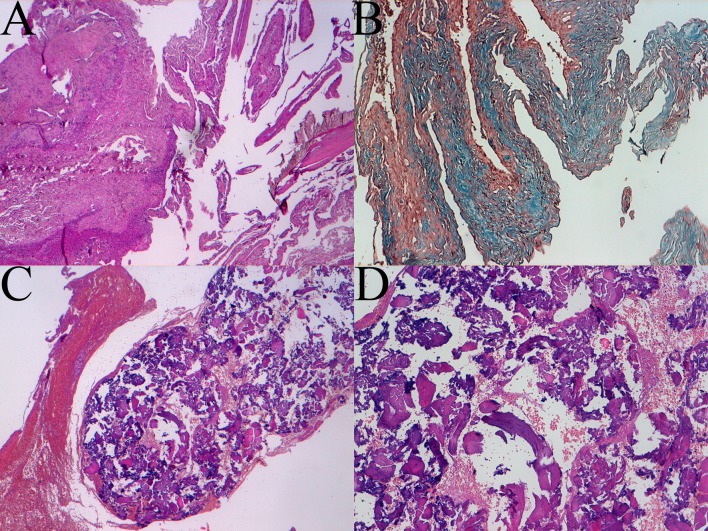
Histologic patterns of meningioangiomatosis: A sclerotic areas (4 × magnification), B trichrome coloration view (10 × magnification), C and D bone metaplasia at 4 × and 10 × magnification respectively.

Histological examination showed areas of collagen "dura-like" (clearly visible after staining with Masson's trichrome) accompanied by areas of bone metaplasia and presence of cerebral gray matter with normal architecture. There was a rich vascularization with vessels often with wall hyalinization, perivascular fibroblastic spindle cells and rare psammoma bodies. Coexisting areas, including extensive benign MA proliferation (EMA +), especially in the perivascular site, were present (Fig. 3).

**Figure 4:**
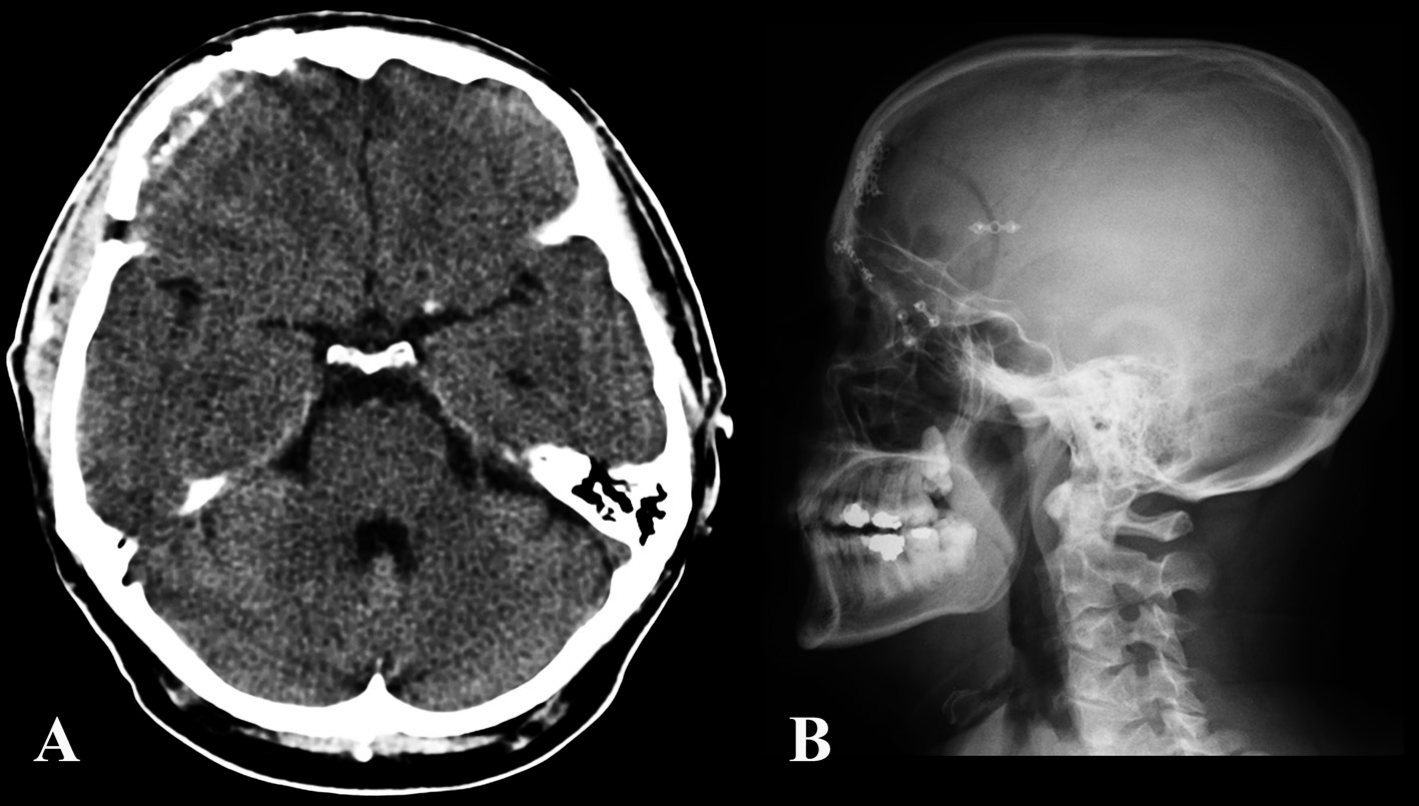
Post-operative CT (A) and skull x-plain film (B) showing the craniotomy and bony reconstruction.

**Figure 5:**
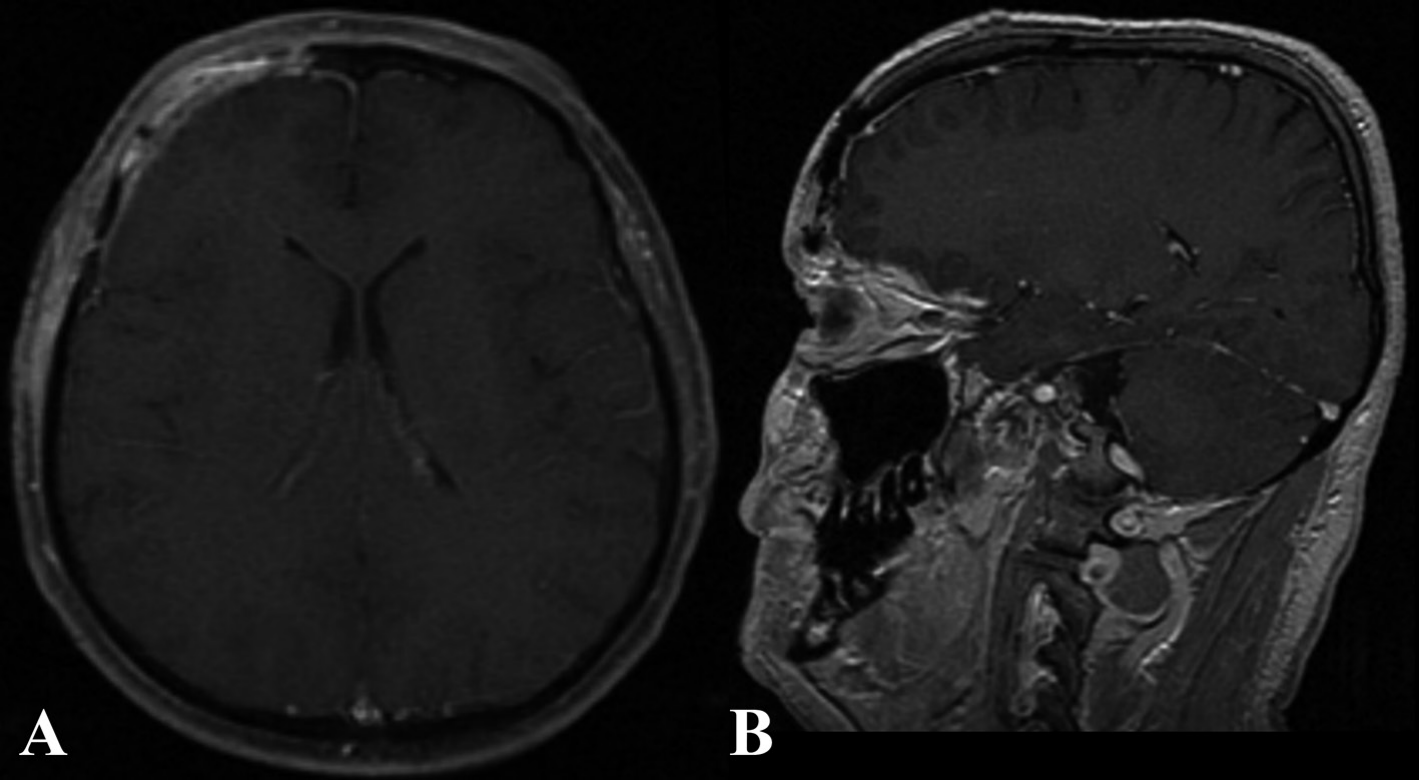
Six months post-operative enhanced MR in the axial (A) and sagittal (B) plain.

The patient’s postoperative course was uneventful. At 6 months follow up, the patient reported no more episodes of headache (Fig. 4, 5).

## Discussion

MA is a rare condition in the adult patients. Only 18 reports with a total of 23 adult patients have been described in literature [1, 2, 4-19]. The average age of the patients reported is 35 years (range 18 - 71) with a relative male prevalence (14:9). The predominant presenting symptoms are seizures (18 cases) and headache (3 cases) but in 2 cases drowsiness and weakness are respectively the initial symptoms. The association with NF2 was described in only 1 case (Table 1).

**Table 1:** Reviewing of the Literature of Meningioangiomatosis in the Adult Patients

Authors	Cases	Age (years)/sex	Localization	NF2	Symptoms
Halper 1986	2	26/m	left temporal	no	seizures
		70/m	left parieto-occipital	no	seizures
Kunishio 1987	1	39/m	right parietal	no	seizures
Sakaki 1987	1	30/f	right temporal	no	seizures
Huson 1988	1	43/m	right frontal	no	drowsiness
Kuzniecky 1988	1	24/m	right temporal	no	seizures
Matias-Guiu 1988	1	42/m	right parietal	no	seizures
Liu 1989	1	25/f	left frontal	no	seizures
Whiting 1990	1	18/f	right temporal	no	seizures
Aizpuru 1991	1	21/m	right temporal	no	-
Prayson 1995	1	32/f	left frontal	no	seizures
Tacconi 1997	1	60/m	right parietal	no	seizures
Chakrabarty 1999	1	22/m	left frontal	no	seizures
Giangaspero 1999	1	28/m	left temporal	no	-
Park 1999	2	47/f	left fronto-parietal	no	headache, seizures
		53/m	left parietal	no	headache, seizures
Wiebe 1999	4	18/f	right occipital	no	seizures
		21/m	left temporal	no	seizures
		36/f	right temporal	no	seizures
		33/f	left temporal	no	seizures
Mut 2000	1	20/f	right temporal	no	seizures
Savargaonkar 2003	1	71/m	both occipital	yes	headache, change in vision
Chen Y 2010	1	34/m	right fronto-parietal	no	weakness, numbness

Generally, MA occurs both in sporadic and syndromic forms, the latter with NF2 [2]. Most cases are asymptomatic; the symptomatic patients tend to present during childhood and early adulthood, usually with seizures. The symptoms are correlated with features of increased intracranial pressure [16]. The tumor may be located in frontal, temporal or parietal cortex with some reports in the third ventricle, cingulate gyrus, pulvinar and brainstem [2, 20].

It was first described by Bassoe and Nuzum in 1915 [21] as an incidental autopsy in a 15-year old boy originally described in association with von Recklinghausen’s disease but it was named by Worster-Drought in 1937 [22]. It is a rare benign disorder characterized by the histological hallmarks of meningiomas and angiomas [4]. In the past, the pathogenesis and histopathological pleomorphism has created diagnostic difficulties [18]. Formerly, MA was considered as a hamartomatous lesion with degenerative changes [4, 21]. Kasanticul and Brown [23] suggested a direct invasion of brain tissue from a leptomeningeal meningioma. Other theories include cortical vascular malformation [24, 25], or the initial development of angiomatous tissue with meningioangiomatous components arising secondarily from perivascular elements [16]. However, even though some studies revealed the loss of 22q12 (NF2 gene) in cases of MA and MA- associated meningioma indicating the possibility of a neoplastic nature [4, 24, 26, 27], a large genetic series demonstrated that there was no NF2 gene mutation either in sporadic or NF2-associated MA in according with the hypothesis of a hamartomatous nature [27, 28].

Generally, solitary MA lesions measuring up to 5 cm are accompanied by meningeal thickening and calcification producing a tan-yellow and gritty tissue [29]. The tumor is usually composed of two components: a leptomeningeal meningothelial proliferation and a meningo-vascular proliferation. The intraparenchymal proliferation of small vessels is accompanied by concentric perivascular proliferation of spindle-shaped meningeal cells similar to those found in a meningioma [20]. The overlying leptomeningeal components of proliferating meningothelial cells can produce marked degenerative reactions such as gliosis, perivascular connective tissue proliferation, dysplastic neurons, white matter cysts and large-vessel hyalinization, calcification, psammoma bodies, fibrocartilage and bone formations [18, 20]. In many cases, proliferating perivascular cells infiltrate the cortex in association with marked cellularity and reactive gliosis [18]. For these reasons, MA can be divided into 3 types: predominantly cellular type, vascular type, and fibrocalcifying type [29]. On the contrary, the presence of neurofibrillary tangles with or without amyloid plaques and granulo-vacuolar degeneration represent a reactive phenomenon rather than an intrinsic lesion component [18].

Although the pre-operative evaluation results difficult because of diagnostic tools lack specificity can be found elements for a correct diagnosis. On the CT scan, these lesions can underline the presence of calcification with surrounding low-density edema. On MRI, they exhibited central low- or mixed-signal intensity on T1-weighted images and a surrounding high-signal edema on T2-weighted images. The enhancement after administration of Gadolinium was seen not in all cases, varying from slight to strong heterogeneous enhancement or even no enhancement at all [14, 18, 30, 31]. It seemed that enhancement patterns had correlation with the proliferating leptomeningeal microvessels [31]. However, MRI can be useful to demonstrate the cortical location of MA, associated with gyriform hyperintensity on FLAIR sequence, which correlated with proliferating microvessels with perivascular cuffs of spindle-cell proliferation within the cortex [31].

The electrophysiological evaluation of MA with and without seizures is complex to analyze with a wide array of findings. Generally, the lesion shows a range of electrophysiological abnormalities. It can encompass circumscribed background slowing and epileptogenicity, multifocal extralesional spikes and sharp waves, and generalized spike-waves without definitive secondary bilateral synchrony or shows an independent and synchronous epileptiform discharges on the surface and within (depth) the MA but not in the adjacent neocortex. However, it can be also electrographically quiescent [18, 19].

Total surgical removal is the treatment of choice for MA. Normally, in these cases the lesion is not growing again and the symptoms usually disappear as occurred in our experience [15, 17, 32]. However in literature seizure-free rates after resection are variably between 43 - 68% of the cases and almost 70 - 80% of the patients continued to required antiepileptic drugs [32, 33].

These results probably arise from a partial surgical resection. In fact, the lesion can extend into the perivascular space but spares the white matter with cortical neurons and rare neurofibrillary tangles entrapped within the lesion [33, 34]. However, the absence of pleomorphism, mitoses, necrosis, cortical invasions or calcifications, that are often associated with MA, cannot allow the complete surgical resection.

In conclusion, MA enters into differential diagnosis with several other diseases such as oligodendroglioma, granulomatous meningitis, meningioma, vascular lesions such as Sturge-Weber syndrome and arteriovenous malformation and parasitic diseases. A correct diagnosis is mandatory because it is a benign lesion that does not become malignant. The total surgical removal is the treatment of choice, and the prognosis after surgery is usually excellent for the absence of recurrence in sporadic cases.
